# Wave-Based Outcomes Comparison of Hospitalized COVID-19 Patients: A Retrospective Multicenter Cohort Study From Rural Appalachia

**DOI:** 10.7759/cureus.51845

**Published:** 2024-01-08

**Authors:** Sandhya Kolagatla, Joshua K Jenkins, Joseph Elsoueidi, Lauren Wisnieski, Nagabhishek Moka

**Affiliations:** 1 Department of Internal Medicine, Appalachian Regional Healthcare, Whitesburg, USA; 2 Department of Surgery, University of Kentucky College of Medicine, Lexington, USA; 3 Department of Hematology and Oncology, University of Kentucky College of Medicine, Lexington, USA; 4 Center for Animal and Human Health in Appalachia, Richard A. Gillespie College of Veterinary Medicine, Lincoln Memorial University, Harrogate, USA; 5 Department of Hematology and Oncology, Appalachian Regional Healthcare, Whitesburg, USA

**Keywords:** kentucky, rural health, outcomes, covid-19, appalachia

## Abstract

Background: There has been little to no characterization of the pandemic’s effects on rural Central Appalachia, in which health disparities in the pre-COVID-19 era have historically plagued. This is the first study to compare wave-based differences in outcomes of hospitalized patients with COVID-19 in the rural Appalachian region. This study aims to provide a more comprehensive understanding of the effects of the COVID-19 pandemic on large rural communities and Appalachia.

Methods: This is a retrospective cohort study of hospitalized patients with COVID-19 between April 2020 and June 2022, which includes 13 Appalachian Regional Healthcare (ARH) hospitals. The primary outcome of the study was in-hospital mortality. Secondary outcomes included intensive care unit (ICU) stay, need for mechanical ventilation, length of hospital stay, 1-30-day re-admittance, 30-60-day re-admittance, and thromboembolism incidence risk.

Results: The second wave of infections during the pandemic demonstrated the highest mortality with higher odds of affecting younger patients. The third wave demonstrated similar mortality to the first wave. Elderly patients and patients with chronic morbidities demonstrated the highest mortality and morbidity and the highest requirement for mechanical ventilation across the three waves. Vaccination lowered the odds of mechanical ventilation and ICU stay.

Conclusions: This study comprehensively characterizes the impact of the COVID-19 pandemic in rural regions of Appalachian Kentucky and West Virginia. Future studies comparing differences between rural and urban geographies may be able to distinguish whether the disparities in these regions played a role in the impact on residents.

## Introduction

SARS-CoV-2, the virus that caused the coronavirus disease 2019 (COVID-19) pandemic, has resulted in 6.8 million deaths across the globe and 2.9 million deaths in the Americas as of March 21, 2023 [1]. At this point in the pandemic, more than 13 billion COVID-19 vaccine doses have been administered worldwide [1]. Throughout the pandemic, cases and hospitalizations related to COVID-19 surged in waves worldwide. The first wave, which began in March-April 2020, severely disturbed social and economic activities due to high mortality and transmissibility worldwide. The second wave began around July 2020 and led to higher mortality rates than the previous wave, and the third wave around January 2021 brought a new strain (B.1.1.7; Delta) with higher transmission rates and increased the number of re-infections [2].

Throughout these waves of the pandemic, rural communities were disproportionately affected compared to their urban dweller counterparts. The origin of their high-risk status is multifactorial, primarily involving a combination of aging populations, multiple comorbidities, and health-related behaviors. For instance, individuals living in rural America are older, with a median age of 51 years compared to 45 years in urban America [3]. Rural communities also have a larger density of residents aged 65 years or older than urban areas (18.4% compared to 14.5%) [4]. Declining birth rates and migration patterns in younger adults have also increased the density of the rural older population more quickly than in urban settings [5]. Rural Americans are more likely to have comorbid health conditions [6,7], limited access to emergency and intensive care healthcare facilities, and live further from healthcare facilities compared to individuals in urban settings [8]. There is also a shortage of healthcare providers in rural America [9]. Health behaviors also put rural populations at risk with higher rates of cigarette smoking, obesity, and physical inactivity compared to more urban populations [10].

Central Appalachia is a unique rural community that endured a significant impact during each wave of the pandemic, regardless of dominant strain, resulting in significant morbidity and mortality. We performed a multicenter retrospective cohort study to evaluate the differences in mortality and morbidity of hospitalized COVID-19 patients in a wave-based distribution. This is the first study in the literature that evaluates wave-based outcomes of hospitalized patients in Central Appalachia.

## Materials and methods

This is a multicenter, retrospective, three-arm cohort study of hospitalized patients with COVID-19 between April 2020 and June 2022, which aims to compare the differences in wave-based outcomes in hospitalized COVID patients. The Appalachian Regional Healthcare (ARH) Institutional Review Board (IRB) approved this study. As per IRB, written consent was waived for this study.

As this is a retrospective study, the data was extracted from electronic medical records (EMRs). Data was extracted from 13 ARH hospitals located in eastern Kentucky and West Virginia. We queried the EMR database for comorbid conditions using the International Classification of Diseases, 10th Revision (ICD-10) master codes. Stata version 17.0 (StataCorp, College Station, TX) was used for all statistical analyses. Bar charts and forest plots were created using the "ggplot2" and "forestplot" packages from R version 4.2.0 (R Foundation for Statistical Computing, Vienna, Austria), respectively [11,12].

Three waves of COVID-19 were defined based on visually assessing a histogram of cases in the hospital system during the study period. The first wave was defined from April 2020 to May 2021, the second wave was defined from June 2021 to November 2021, and the third wave was defined from December 2021 to June 2022. The primary outcome of the study was in-hospital mortality, whereas the secondary outcomes included intensive care unit (ICU) stay, need for mechanical ventilation, length of hospital stay (LOS), 1-30-day re-admittance, 30-60-day re-admittance, and thromboembolism incidence risk.

Data analysis

Descriptive and chi-square analyses were used to compare demographic characteristics, comorbidities, and discharge status between waves. Analysis of variance (ANOVA) was used to compare the age and body mass index (BMI) of patients between waves. 

Outcomes among COVID-19 patients between the three waves were compared using adjusted and unadjusted regression models. Mixed-effects logistic regression using the "melogit" command in Stata was used to model mortality, ICU stay, mechanical ventilation, 1-30-day re-admittance, 31-60 day re-admittance, and thromboembolism risk. Mixed linear regression using the "xtmixed" command in Stata was used to model LOS. A random intercept for hospital (N=13) was included in all models. Adjusted analyses were adjusted for age, gender, marital status, obesity status, diabetes, hypertension, pulmonary disease, chronic kidney disease, autoimmune disease, coronary heart disease, and tobacco use. An interaction term between age category and wave was assessed in each model to determine if trends in the likelihood of different outcomes changed across each age category between the three waves. 

Normality and homoskedasticity of residuals were assessed using residual plots (i.e., histograms and quantile-quantile [Q-Q] plots) for mixed linear regression models and ANOVA tests. LOS and BMI were transformed by the logarithm function to meet normality requirements. All model results for the transformed variables were back-transformed to improve interpretability.

## Results

**Figure 1:**
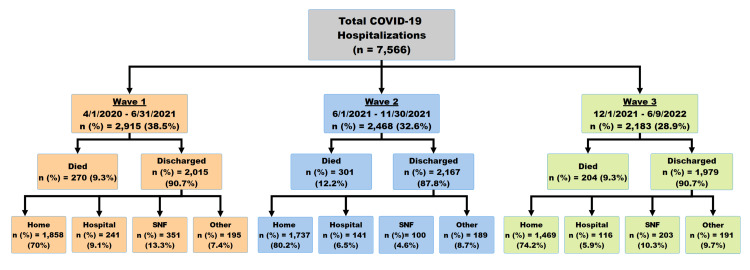
Summary of patients hospitalized with COVID-19 during the COVID-19 pandemic.

In total, data from 7,572 patients was downloaded from the EMR database. Six records were missing discharge data and were removed, leaving a total of 7,566 records (Figure 1). There was missing data at the patient level for BMI (n=300) and marital status (n=71).

Patients in wave 2 had a higher mortality rate (12.2%) compared with those in wave 1 (9.3%) and wave 3 (9.3%). During wave 1, patients were older on average (64.2 years) compared to wave 2 (58.8 years) and wave 3 (62.5 years). A larger percentage of patients were married in wave 1 (50.0%) and wave 2 (49.2%) compared to wave 3 (43.1%) (Table 1). In addition, the percentage of female patients was higher in wave 1 (50.8%) and wave 2 (48.7%) compared to wave 3 (45.1%). The average BMI was lower among those in wave 3 (30.7) versus wave 1 (32.0) and wave 2 (32.1). Other differences in the prevalence of comorbidities between waves are presented in Table 1. 

**Table 1:** Characteristics of a sample of hospitalized COVID-19 patients stratified by wave (N=7,566) CHF, congestive heart failure; TIA, transient ischemic attack; ICU, intensive care unit.

		Wave 1 (n=2,915)		Wave 2 (n=2,468)		Wave 3 (n=2,183)		Total (n=7,566)		
Characteristic		n	% (within row)	n	% (within row)	n	% (within row)	n	% (of total)	p-Value
Demographics										
Age (years)	18 to 39	238	29.13	340	41.62	239	29.25	817	10.8	<0.001
	40 to 49	310	35.35	349	39.79	218	24.86	877	11.59	-
	50 to 64	766	35.04	794	36.32	626	28.64	2,186	28.89	-
	65 to 74	762	40.55	558	29.7	559	29.75	1,879	24.83	-
	74 and older	839	46.43	427	23.63	541	29.94	1,807	23.88	-
Marital status	Married	1,443	40.31	1,204	33.63	933	26.06	3,580	47.77	<0.001
	Single	546	35.09	534	34.32	476	30.59	1,556	20.76	-
	Divorced/separated/widowed	897	38.02	708	30.01	754	31.96	2,359	31.47	<0.001
Gender	Male	1,480	40.36	1,202	32.78	985	26.86	3,667	48.47	-
	Female	1,435	36.8	1,266	32.47	1,198	30.73	3,899	51.53	-
Insurance	Medicaid/Medicare	2,313	37.73	1,967	32.09	1,850	30.18	6,130	81.02	<0.001
	Other	602	41.92	501	34.89	333	23.19	1,436	18.98	-
Comorbidities										
Weight status	Underweight	85	33.46	66	25.98	103	40.55	254	3.5	<0.001
	Normal weight	500	35.46	443	31.42	467	33.12	1,410	19.41	-
	Overweight	747	38.91	606	31.56	567	29.53	1,920	26.42	-
	Obese	570	38.2	517	34.65	405	27.14	1,492	20.53	-
	Morbidly obese	882	40.27	753	34.38	555	25.34	2,190	30.14	-
Diabetes	No	1,807	36.68	1,651	33.51	1,469	29.82	4,927	65.12	<0.001
	Yes	1,108	41.99	817	30.96	714	27.06	2,639	34.88	-
Hypertension	No	1,829	37.69	1,533	31.59	1,491	30.72	4,853	64.14	<0.001
	Yes	1,086	40.03	935	34.46	692	25.51	2,713	35.86	-
Hepatitis	No	410	38.68	342	32.26	308	29.06	1060	91.7	0.03
	Yes	26	27.08	43	44.79	27	28.13	96	8.3	-
Pulmonary disease	No	2,842	38.79	2,380	32.49	2,104	28.72	7,326	96.83	0.03
	Yes	73	30.42	88	36.67	79	32.92	240	3.17	-
Kidney disease	No	2,354	37.67	2,130	34.09	1,765	28.24	6,249	82.59	<0.001
	Yes	561	42.6	338	25.66	418	31.74	1,317	17.41	-
Kidney failure	No	2,211	39.3	1,830	32.53	1,585	28.17	5,626	74.36	0.03
	Yes	704	36.29	638	32.89	598	30.82	1,940	25.64	-
Malignancy	No	2,799	38.41	2,391	32.81	2,097	28.78	7,287	96.31	0.19
	Yes	116	41.58	77	27.6	86	30.82	279	279	-
Acute respiratory failure	No	1,744	40.13	1,281	29.48	1,321	30.4	4,346	57.44	<0.001
	Yes	1,171	36.37	1,187	36.86	862	26.77	3,220	42.56	-
CHF	No	2,404	38.37	2,107	33.63	1,754	28	6,265	82.8	<0.001
	Yes	511	39.28	361	27.75	429	32.97	1,301	17.2	-
Dementia	No	2,691	37.72	2,387	33.45	2,057	28.83	7,135	94.3	<0.001
	Yes	224	51.97	81	18.79	126	29.23	431	5.7	-
Osteoarthritis	No	2,617	38.03	2,272	33.01	1,993	28.96	6,882	90.96	0.01
	Yes	298	43.57	196	28.65	190	27.78	684	9.04	-
Obstructive pulmonary disease	No	2,178	38.03	1,918	33.49	1,631	28.48	5,727	75.69	0.02
	Yes	737	40.08	550	29.91	552	30.02	1,839	24.31	-
Pulmonary embolism	No	2,888	38.56	2,453	32.75	2,148	28.68	7,489	98.98	0.003
	Yes	27	35.06	15	19.48	35	45.45	77	1.02	-
Lipidemia	No	1,937	36.73	1,780	33.75	1,557	29.52	5,274	69.71	<0.001
	Yes	978	42.67	688	30.02	626	27.31	2,292	30.29	-
History of TIA and cerebral infarction	No	2,756	38.53	2,345	32.79	2,051	28.68	7,152	94.53	0.28
	Yes	159	38.41	123	29.71	132	31.88	414	5.47	-
Severe sepsis with shock	No	2,784	38.58	2,371	32.86	2,061	28.56	7,216	95.37	0.03
	Yes	131	37.43	97	27.71	122	34.86	350	4.63	-
Autoimmune disorder	No	2,122	37.59	1,891	33.5	1,632	28.91	5,645	74.61	0.01
	Yes	793	41.28	577	30.04	551	28.68	1,921	25.39	-
Tobacco use	No	2,551	40.63	2,020	32.18	1,707	27.19	6,278	82.98	<0.001
	Yes	364	28.26	448	34.78	476	36.96	1,288	17.02	-
Long-term opiate use	No	2,801	38.65	2,359	32.55	2,087	28.8	7,247	95.78	0.58
	Yes	114	35.74	109	34.17	96	30.09	319	4.22	-
COVID-19 vaccination	No	1,903	37.45	1,839	36.19	1,339	26.35	5,081	67.16	<0.001
	Yes	1,012	40.72	629	25.31	844	33.96	2,485	32.84	-
Outcomes										
ICU	No	2,537	38.99	2,105	32.35	1,864	28.65	6,506	85.99	0.12
	Yes	378	35.66	363	34.25	319	30.09	1,060	14.01	-
Mortality	No	2,645	38.95	2,167	31.91	1,979	29.14	6,791	89.76	<0.001
	Yes	270	34.84	301	38.84	204	26.32	775	10.24	-
1-30-day re-admit	No	2,132	38.21	1,822	32.66	1,625	29.13	5,579	82.15	0.01
	Yes	513	42.33	345	28.47	354	29.21	1,212	17.85	-
31-60-day re-admit	No	2,434	39.13	2,008	32.28	1,778	28.59	6,220	91.59	0.003
	Yes	211	36.95	159	27.85	201	35.2	571	8.41	-
Mechanical ventilation	No	2,367	38.82	1,926	31.59	1,804	29.59	6,097	80.58	<0.001
	Yes	548	37.3	542	36.9	379	25.8	1,469	19.42	-
Clot risk	No	2,819	38.79	2,369	32.6	2,079	28.61	7,267	96.05	0.03
	Yes	96	32.11	99	33.11	104	34.78	299	3.95	-

Outcomes by wave

**Figure 2:**
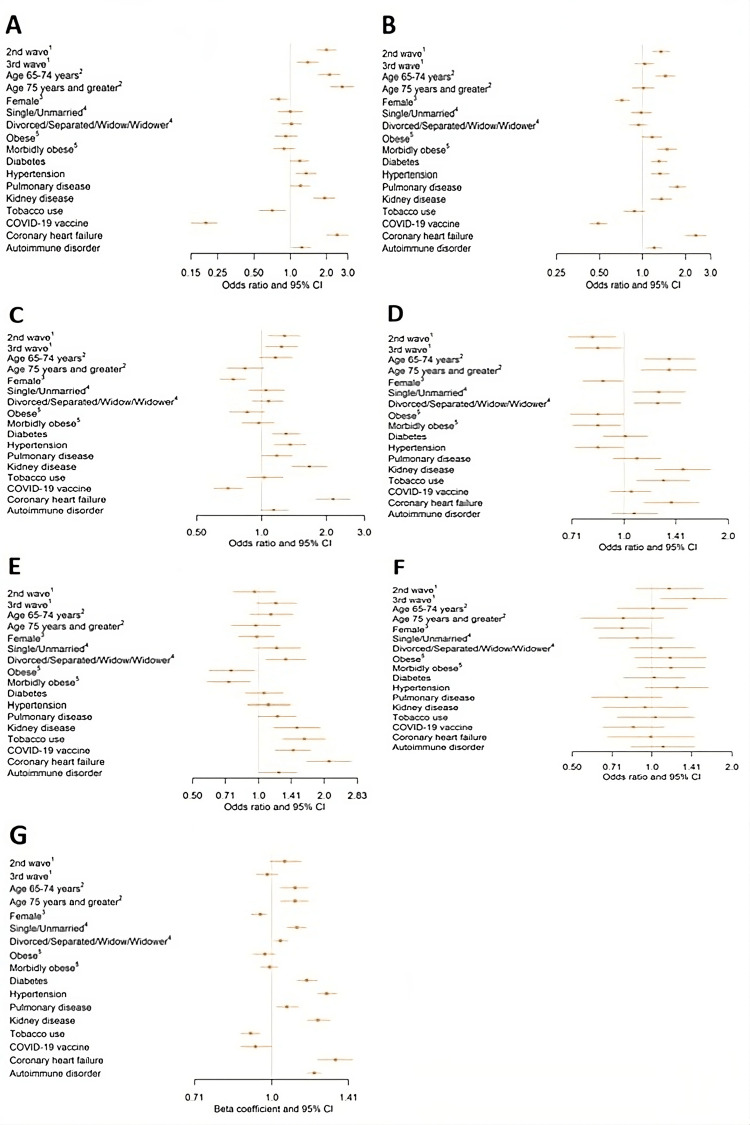
Demonstrates odds ratios for (A) mortality, (B) mechanical ventilation requirement, (C) ICU stay requirement, (D) 1-30-day re-admittance, (E) 31-60-day re-admittance, and (F) being at high risk for development of venous thromboembolism in a sample of patients with primary or secondary diagnoses of COVID-19 by demographic group and comorbidity (N = 7,197). In reference to these figures, 1 is in reference to the first wave of infections, 2 is in reference to ages <65 years, 3 is in reference to the male sex, 4 is in reference to a married marital status, and 5 is in reference to a Not Obese classification by BMI. Figure 2G represents the factors associated with LOS in the same sample of patients. In this figure, coefficients are back-transformed from the logarithm scale. Coefficients <1 are associated with a shorter LOS.

In adjusted and unadjusted analyses, the odds of death and requiring mechanical ventilation were lowest during wave 1, followed by wave 3, and were highest in wave 2 (Table 2). Patients in wave 2 had greater than two times the odds of mortality compared to patients in wave 1 in adjusted analyses (OR [95% CI]: 2.44 [1.71-3.49]; Table 2; Figure 2A). In adjusted analyses, odds of ICU stay were also higher in wave 2 (OR [95% CI]: 1.28 [1.08-1.51]) and wave 3 (OR [95% CI]: 1.24 [1.04-1.48]) in comparison to wave 1 (Table 2; Figure 2C). In both unadjusted and adjusted analyses, the odds of re-admittance between 1 and 30 days were approximately 20% lower in wave 2 (OR [95% CI]: 0.81 [0.69-0.95]) and wave 3 (OR [95% CI]: 0.84 [0.72-0.98]) compared to wave 1. However, the odds of re-admittance between 31 and 60 days during wave 3 were higher than wave 1 and wave 2, although this was only significant in unadjusted analyses. The odds of thromboembolism risk were highest in wave 3, although this was also not statistically significant in adjusted analyses. Finally, in unadjusted analyses, LOS was shorter in wave 3 compared to wave 1 and wave 2, although this was not statistically significant in adjusted analyses. 

**Table 2:** Adjusted and unadjusted results for the association of COVID-19 diagnosis and multiple outcomes in a sample of hospitalized CHF patients (N = 7,566). CHF, congestive heart failure. *p ≤ 0.05, when compared to the reference level. ^1^Adjusted for age, gender, marital status, obesity status, diabetes, hypertension, pulmonary disease, kidney disease, autoimmune disease, coronary heart disease, and tobacco use. ^2^Estimates are back-transformed from the logarithmic scale.

	Unadjusted OR (95% CI)	Adjusted OR (95% CI)^1^
Mortality		
Wave 1 (reference)	1	1
Wave 2	1.40 (1.18-1.67)*	2.00 (1.64-2.44)*
Wave 3	1.05 (0.87-1.27)*	1.40 (1.13-1.73)*
Mechanical ventilation		
Wave 1 (reference)	1	1
Wave 2	1.24 (1.08-1.42)*	1.35 (1.17-1.56)*
Wave 3	0.92 (0.80-1.07)	1.04 (0.88-1.21)
ICU stay		
Wave 1 (reference)	1	1
Wave 2	1.20 (1.03-1.41)*	1.28 (1.08-1.51)*
Wave 3	1.19 (1.01-1.40)*	1.24 (1.04-1.48)*
1-30 d readmittance		
Wave 1 (reference)	1	1
Wave 2	0.77 (0.66-0.89)*	0.81 (0.69-0.95)*
Wave 3	0.90 (0.77-1.05)	0.84 (0.72-0.98)*
31-60 d readmittance		
Wave 1 (reference)	1	1
Wave 2	0.91 (0.74-1.13)	0.96 (0.77-1.20)
Wave 3	1.32 (1.08-1.62)*	1.20 (0.97-1.49)
Clot risk		
Wave 1 (reference)	1	1
Wave 2	1.25 (0.94-1.67)	1.16 (0.87-1.56)
Wave 3	1.43 (1.08-1.90)*	1.44 (1.07-1.92)*
Length of stay in days^2^		
Wave 1 (reference)	1	1
Wave 2	1.00 (0.92-1.09)	1.06 (0.99-1.03)
Wave 3	0.93 (0.89-0.98)	0.98 (0.93-1.03)

Comorbidities 

The odds of mortality increased by age group (Figure 2A). Patients who were 75 years or older had greater than two times the odds of mortality compared with those patients aged 18-64 years (OR [95% CI]: 2.70 [2.14-3.40]). Females had lower odds of mortality compared with males (OR [95% CI]: 0.80 [0.68-0.95]). Comorbidities associated with greater odds of mortality included diabetes (OR [95% CI]: 1.20 [1.00-1.43]), hypertension (OR [95% CI]: 1.36 [1.11-1.67]), pulmonary disease (OR [95% CI]: 1.22 [1.01-1.46]), chronic kidney disease (OR [95% CI]: 1.93 [1.56-2.39]), coronary heart disease (OR [95% CI]: 2.45 [1.99-3.03]), and autoimmune disease (OR [95% CI]: 1.25 [1.04-1.49]). Having a COVID-19 vaccine was associated with 80% lower odds of mortality (OR [95% CI]: 0.20 [0.15-0.25]). Interestingly, tobacco use was also associated with lower odds of mortality (OR [95% CI]: 0.71 [0.55-0.92]). 

The odds of mechanical ventilation were highest among those aged 64-74 years (Figure 2B). Patients aged less than 65 and 75 years and older had similar odds of mechanical ventilation. Risk factors for mechanical ventilation included morbid obesity (OR [95% CI]: 1.49 [1.28-1.74], compared to not being obese), diabetes (OR [95% CI]: 1.31 [1.15-1.50]), hypertension (OR [95% CI]: 1.33 [1.15-1.55]), chronic kidney disease (OR [95% CI]: 1.36 [1.14-1.62]), coronary heart disease (OR [95% CI]: 2.37 [2.00-2.81]), and having an autoimmune disorder (OR [95% CI]: 1.21 [1.06-1.39]). Females had lower odds of requiring mechanical ventilation (OR [95% CI]: 0.72 [0.64-0.82]). Those who received the COVID-19 vaccination had 51% lower odds of requiring mechanical ventilation compared with those who did not (OR [95% CI]: 0.49 [0.43-0.57]). 

Risk factors for ICU stay included diabetes (OR [95% CI]: 1.30 [1.12-1.51]), hypertension (OR [95% CI]: 1.36 [1.14-1.61]), pulmonary disease (OR [95% CI]: 1.18 [1.01-1.39]), kidney disease (OR [95% CI]: 1.67 [1.38-2.02]), and coronary artery disease (OR [95% CI]: 2.15 [1.78-2.59]) (Figure 2C). Being female was associated with lower odds of ICU stay (OR [95% CI]: 0.74 [0.64-0.85]). Having the COVID-19 vaccination was also associated with lower odds of ICU stay (OR [95% CI]: 0.70 [0.60-0.82]). 

**Figure 3:**
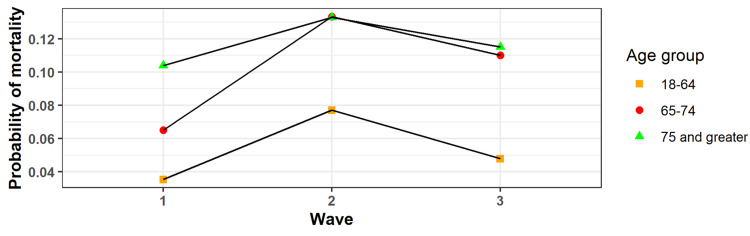
Displays the probability of mortality in each wave stratified by age categorization.

Older age (>64 years old) and not being married were associated with about 30% greater odds of being re-admitted within 1-30 days (Figure 3A). Other risk factors for re-admittance between 1 and 30 days included chronic kidney disease (OR [95% CI]: 1.48 [1.23-1.78]), tobacco use (OR [95% CI]: 1.30 [1.09-1.55]), and coronary artery disease (OR [95% CI]: 1.37 [1.14-1.65]). Females had lower odds of being re-admitted within 1-30 days (OR [95% CI]: 0.87 [0.76-0.99]). Interestingly, obese patients and those with the COVID-19 vaccine had higher odds of re-admission. In addition, patients with hypertension had lower odds of being re-admitted (OR [95% CI]: 0.84 [0.72-0.99]).

Risk factors for being re-admitted within 31-60 days included being divorced, separated, or widowed (OR [95% CI]: 1.33 [1.08-1.65], compared to being married) (Figure 2E), chronic kidney disease (OR [95% CI]: 1.50 [1.17-1.91]), tobacco use (OR [95% CI]: 1.62 [1.29-2.03]), coronary artery disease (OR [95% CI]: 2.10 [1.65-2.67]), having the COVID-19 vaccine (OR [95% CI]: 1.44 [1.19-1.73]), and autoimmune disease (OR [95% CI]: 1.24 [1.01-1.51]). Obesity lowered the odds of being re-admitted. 

The only factor associated with high thromboembolism risk was gender (Figure 2F). Females had lower odds of being at high risk for thromboembolism compared to males (OR [95% CI]: 0.77 [0.60-0.98]).

Demographics associated with a longer LOS included being older (>64 years old) and unmarried. Both of these factors increased the average LOS by approximately one day (Figure 2G). Other factors associated with a longer LOS include diabetes, hypertension, obstructive pulmonary disease, chronic kidney disease, coronary artery disease, and autoimmune disease. Being female, using tobacco, and having the COVID-19 vaccine were associated with a shorter LOS. 

Age and wave interaction effects 

The only outcome with a significant interaction effect between the age group and the wave was mortality (Figure 3). Across all age groups, mortality was highest in wave 2. However, those in the 65-74-year age group experienced a larger increase in mortality from wave 1 to wave 2. 

## Discussion

To our knowledge, this is the first study to compare the wave-based differences in outcomes of hospitalized patients with COVID-19 in the rural Appalachian region. This study offers a more comprehensive characterization of the effects of the COVID-19 pandemic on patients in the rural regions of Appalachian Kentucky and West Virginia.

Though exact strains were not isolated and reported for this study, the data gives indirect insight into the virulence patterns of the different COVID-19 strains most predominant in the country during these waves and how they may have affected rural Appalachian communities. For instance, the SARS-CoV-2 B.1.617.2 (Delta) variant emerged in the United States in June 2021 and remained the predominant sequenced lineage between July 2021 and November 2021 [13]. This correlates with the timeline of our second wave of infections in Appalachian Kentucky and West Virginia. The national Delta period was characterized by the highest crude mortality risk (15.1) and adjusted mortality risk for patients older than or equal to 18 years [14]. This is consistent with our data characterizing the second wave of infections, which also demonstrated the highest odds of mortality across all age groups, mechanical ventilation requirement, and ICU stay requirement in the region. The SARS-CoV-2 B.1.1.529 (Omicron) variant emerged in the United States in December 2021 and accounted for 72% of sequenced lineages by December 25, 2021 [15]. This emergence correlates with our third wave of infections in Appalachian Kentucky and West Virginia. The national Omicron period was characterized by a chronological decrease in crude mortality rate (13.1 in the early period; 4.9 in the late period), with 81.9% of in-hospital deaths occurring in elderly adults aged 65 years or older and 73.4% of in-hospital deaths occurring in patients with three or more underlying medical conditions [14]. This is also consistent with our data from the region, which demonstrated a similar mortality rate in the third wave compared to the first wave of infections. 29.8% of patients in wave 3 were aged 65 years or older, whereas 26.7% were in the same age group in wave 2. The first wave of infections in the region was likely a combination of the remaining major viral variants, including B.1.1.7 (Alpha), B.1.351 (Beta), and/or P.1 (Gamma), as these strains were most predominant in the timeline of wave 1 (April 2022 to May 2021) [16].

The increased odds of mortality, odds of ICU admittance, and odds of longer LOS demonstrated in wave 2 are likely primarily related to the presumed predominant viral strain according to the aforementioned timelines, which had been compounded in the Appalachian region through a combination of the poor resource allocation and funding in the rural setting during the pandemic. Regarding the strain of healthcare resources in the rural Appalachian setting, public health funding in this region is vastly inefficient. Allocation of healthcare resources is tiered. At the policy level, which is the highest level of resource allocation, strategies are determined through legislation, health insurance plans, and government funding mandates. At the organizational level, allocation decisions are made through institutional policies, clinical practice guidelines, and protocols [17]. For example, limited resources at the organizational level may attempt to maximize resources through triage protocols. Microallocation of resources, the lowest level of resource allocation, is mostly provider-dependent, anchored by the medical decision-making process and assessing risks versus benefit scenarios for particular interventions related to patient care [17].

During the COVID-19 pandemic, macroallocation in the form of federal mandates and legislation shifted the availability of resources as rural health departments rely heavily on state and federal funds. Local public health funding is often determined by a region’s overall wealth and tax base [18]. With rural communities facing a declining tax base and often having lower overall wealth, local health departments are typically left with insufficient or less stable funding. In Kentucky, local and state health officials worked to transform the public health funding model in 2018 to direct more resources to areas with the greatest needs to ensure equitable access to essential services and supplies [18]. This funding model, however, was hampered by budget constraints associated with the pandemic. The budget constraints also exacerbated financial issues at the institutional level in this region. As the virus spread throughout the United States, providers across the country were forced to cancel elective procedures, close or limit primary care and outpatient clinic hours, and shift resources from higher margin care to focus on acute COVID-19 cases, which inevitably led to an institutional loss of revenue [19]. As the Appalachian region was not exempt from this effect, this exacerbated institutional funding problems. In response, many hospitals throughout the pandemic were forced to furlough staff or reduce working hours, leading to staffing constraints within hospitals, role frustration, and burnout [19].

The odds of overall mortality in this region were increased at baseline, given the comorbidity of the patient population. Comorbid conditions, such as smoking status, chronic obstructive pulmonary disease (COPD), diabetes, and obesity, have been deemed clinical risk factors for fatal outcomes associated with coronavirus [20]. In Central Appalachia, the prevalence of smoking (the leading cause of COPD in the United States) in adults is 25.2% in comparison to the national level of 16.3%. The prevalence of diabetes in Appalachia is 11.9% overall (13.7% in the region’s most distressed counties) in comparison to the national mark of 9.8%. Obesity is 34.7% prevalent in the Central Appalachian region, which is also higher than the national level of 27.4% [21]. Concerning the present study, tobacco use, obstructive pulmonary disease, and diabetes were all less present in hospitalized patients. However, obesity in general was present in slightly more than half of hospitalized patients, with a vast majority of patients at least being overweight. These results were surprising; however, it is possible that more critical patients with these comorbidities were admitted to one of the tertiary care institutions in the region instead of the lower acuity hospitals in the ARH healthcare system.

Limitations

This study has several limitations. Recall bias and database documentation errors are derived from the intrinsic study design. Along with these limitations, the predominant COVID-19 strain for each wave in this area of Central Appalachia was not recorded in the EMR database. Therefore, the dominant viral strain per wave is unknown. Racial characterizations for patients were not captured in the datasets for the study. Thus, racial disparities related to the pandemic in rural Central Appalachia could not be measured. Dates were based on the dates of initial admittance to the ARH healthcare system, not the date of symptom onset or diagnosis. Our team could also not assess the differences in severity or stage of comorbid conditions because only ICD-10 master codes were utilized.

Additionally, the use of remdesivir in hospitalized adult patients with COVID-19 has been shown to result in a moderate reduction in length of stay in previous studies [22]. However, dates of administration of remdesivir or steroid therapy were not captured in this database for inclusion in this study. We cannot translate these results on LOS in Appalachia. However, this study aimed to represent the pandemic in a large, underserved region comprehensively. It was not meant to evaluate the relationship of individual therapies in the region. Future studies comparing the clinical impacts of specific variants in rural Appalachian and urban or suburban communities should be assessed to unveil potential health disparities between the communities. Further, retrospective or prospective studies examining the effects of different therapeutic modalities, such as steroid therapy or remdesivir administration, in this particularly comorbid patient population may provide interesting insight.

## Conclusions

This is the first study to compare the wave-based differences in outcomes of hospitalized patients with COVID-19 in the rural Appalachian region. This report characterizes the COVID-19 pandemic’s effect on the Central Appalachian region. The second wave of infections during the pandemic resulted in the highest mortality rate, with elderly patients and patients with comorbidities being the most significant. Future studies comparing the clinical impacts of specific variants in rural Appalachian and urban or suburban communities should be evaluated to unveil potential health disparities between the communities.
